# A Relational Agent Intervention for Adolescents Seeking Mental Health Treatment: Outcomes From a Randomized Controlled Trial Within a Children’s Outpatient Hospital

**DOI:** 10.1016/j.jaacop.2025.02.002

**Published:** 2025-02-11

**Authors:** Mary Margaret Gleason, Megan Flom, Stephanie Rapoport, Andre Williams, Autumn Birch, Nicole K. Wells, Valerie Forman-Hoffman, Athena Robinson

**Affiliations:** a Children’s Hospital of The King's Daughters and Eastern Virginia Medical School, Norfolk, Virginia; b Woebot Health, San Francisco, California

**Keywords:** relational agent, randomized controlled trial, adolescent mental health, digital mental health, noninferiority

## Abstract

**Objective:**

Despite steady increases in the prevalence of depression and anxiety, many adolescents remain undertreated. Digital mental health interventions (DMHI) may address some barriers to care. This study is the first to examine the prospective noninferiority of a smartphone-based, self-guided relational agent (W-GenZD) DMHI to address adolescent depressive symptoms compared to group-based telehealth cognitive-behavioral therapy (CBT).

**Method:**

Adolescents 13 to 17 years of age who were seeking treatment for depression and/or anxiety at a children’s hospital outpatient mental health program and who met study inclusion criteria were randomized to either W-GenZD (n = 71) or CBT Group (n = 70). The primary aim was to examine feasibility and acceptability. The secondary aim was to test the noninferiority of W-GenZD compared to CBT Group on the 8-item Patient Health Questionnaire (PHQ-8) at 4 weeks (end of treatment [EOT]). The study also examined the trajectory of working alliance, anxiety, general mood, and safety in each intervention group.

**Results:**

W-GenZD was noninferior to CBT Group in reducing depressive symptoms, with a mean between-group PHQ-8 EOT score difference of −0.67, 95% CI = − 2.30, 0.82 in intent-to-treat analysis (N = 141) using multiple imputation for missing values. Four different sensitivity analyses confirmed noninferiority, including per protocol analysis (−1.38, −3.61, 0.86, n = 82). Feasibility, acceptability, and tertiary aims were similar in both groups, and data support the safety of W-GenZD in a mental health outpatient setting.

**Conclusion:**

Results suggest that W-GenZD is a feasible and efficacious intervention for adolescents presenting with depression and/or anxiety in a real-world setting. W-GenZD could help expand the capacity of mental health clinicians to support the many youth and families in need of mental health care.

**Clinical trial registration information:**

Feasibility and Acceptability of W-GenZD vs CBT-light Teletherapy for Adolescents Seeking Mental Health Services; https://clinicaltrials.gov/study/NCT05372913

## Plain language summary

Many adolescents with depression and anxiety can’t access professional help. Digital tools, including apps, may help address barriers to care. This is the first study to compare an interactive chat-based app (W-GenZD) with active therapy—in telehealth-delivered group-based cognitive-behavioral therapy (CBT Group)—among adolescents with symptoms of depression and/or anxiety in a real-world children’s hospital outpatient setting. Results suggest that W-GenZD was noninferior to CBT Group in reducing depressive symptoms at 4 weeks or end of treatment. Feasibility and acceptability of the interventions were similar in both groups and data support the safety of W-GenZD in a mental health outpatient setting.

Prevalence rates of pediatric depression and anxiety were steadily rising prior to the COVID-19 pandemic,^1^ with rates of mild and clinically elevated symptomatology increasing exponentially during that time.^2^ Despite the rapid rise among youth, this population has been grossly undertreated.^3^ Less than half of youth with severe symptoms and impairment receive treatment, and rates are even lower for youth with minimal impairment.^4^ Barriers to care are multifactorial, with children and adolescents identifying barriers related to individual factors (eg, limited knowledge about mental health and mental health services), social factors (eg, perceived stigma around help-seeking), relational factors (eg, concerns regarding confidentiality), and systematic and structural factors (eg, limited availability of professional services).^5^

Effective psychotherapeutic approaches to address adolescent depression and anxiety are well established. Cognitive–behavioral therapy (CBT) is a first-line treatment for adolescent depression and anxiety in practice guidelines across mental health specialties.^6^^,^^7^ Pediatric literature supports the effectiveness of CBT across a range of delivery modalities, formats and durations. Recent meta-analytic data found comparable effects between different forms of CBT on youth depression and anxiety, including that delivered in person vs that delivered via telehealth or Internet,^8^^,^^9^ and between individual and group based.^10^^,^^11^ Furthermore, the literature reports efficacy of brief interventions, ranging from 1 to 6 sessions, on adolescent depression and anxiety, and no difference in overall efficacy based on dose of CBT.^12^^,^^13^ Large reviews reveal a positive impact of even single-session interventions for youth anxiety and, to a lesser degree, depression.^12^^,^^14^ Analyses of treatment response trajectories suggest that specific content (self-talk, exposure) may be responsible for most rapid changes in symptoms, and reliable change is seen in fewer than 3 sessions of CBT.^15^ In real-world settings, the average course of CBT is less than 4 sessions, although many models are intended to deliver 12 or even more sessions.^16^ Within the diversity of delivery approaches, empirically supported core factors (eg, rapport, psychoeducation, cognitive restructuring, and exposure) are thought to be critical for clinical effectiveness.^11^

Despite the strength of evidence supporting CBT-based interventions for adolescents with depression and anxiety, effective delivery of CBT is limited by access to trained mental health professionals, the time and resources that traditional therapy attendance requires, a mismatch between traditional office hours and families’ availability, stigma-related hesitation, and limited trust that professionals will be able to help.^5^^,^^17^

Digital mental health interventions (DMHI) may expand the options for low-intensity interventions to support youth with mental health concerns, especially in mental health workforce shortage areas. DMHI that include evidence-based elements offer promise in supporting youth mental health because they offer accessibility, asynchronous interactions, discreet care delivery, and do not require travel, schedule, and logistical coordination often requisite of in-person appointments at brick-and-mortar clinics.^18^ Youth themselves identify ease of use, access, less stigma, and lack of waiting as benefits to mobile- or m-Health resources,^19^ and most report interest in using technologies to support their mental health.^20^

A growing evidence base supports the efficacy of DMHI with effect sizes similar to those of therapist-delivered care.^21^ A recent meta-analysis of 66 randomized controlled trials (RCTs) of app-delivered interventions (primarily for adult mental health care) found that apps outperformed inactive control conditions (ie, waitlist, information-only and/or attention/placebo control) on improving depressive and anxiety symptoms, and other key clinical outcomes, with the strongest effect sizes for apps offering interactive CBT.^22^ In this meta-analysis, apps did not differ significantly from active, face-to-face delivered interventions, although the number of studies comparing apps to active treatment is limited. In addition, effects were consistent among populations who met diagnostic criteria as well as among those whose symptoms were subclinical (eg, general community sample).

Despite DMHI research expanding exponentially, pediatric-focused randomized controlled trials (RCTs) are relatively limited. A few studies have focused on clinical populations, yet did not show improvement in mental health indices compared to controls.^23^^,^^24^ Moreover, most reports of adolescent-focused Web-based or mobile-based programs are curricular in nature or foster communication with a clinician.^25^^,^^26^ Some studies have investigated implementation success rather than clinical effectiveness, with generally positive results.^27^ Although a 2021 systematic review of DMHI RCTs included some studies with participants with a mean age of less than 18 years, none of the included studies examined the effectiveness of relational agents (interactive chatbots) among this age group.^25^ In DMHI fields, a relational agent is an interactive, defined as a conversationally based, character agent designed to establish and sustain rapport or working alliance with its users.^28^ To our knowledge, no existing adolescent RCTs have examined stand-alone DMHI (ie, nonadjunctive to psychotherapy) guided by a relational agent to therapist-delivered care. In addition, no evaluations of such relational agent**–**guided DMHI among adolescents to date have been compared as a stand-alone intervention to another active intervention in an ecologically valid real-world setting.

Woebot Health’s suite of DMHIs are delivered through a smartphone app, in which a relational agent called Woebot offers CBT-guided self-help to users through text-based conversation, with demonstrated preliminary efficacy to reduce symptoms of depression among adults^29^ and adolescents^30^ as well as feasibility, acceptability, and safety.^31^ In addition, Woebot has demonstrated an ability to support a working alliance with both its adult and its adolescent users.^30^^,^^32^ W-GenZD was specifically developed for adolescents 13 to 17 years of age.

This study examined the feasibility and acceptability of W-GenZD among adolescents presenting with anxiety or depression in a real-world clinical practice of a hospital-based outpatient program. The secondary aim was to test the noninferiority of W-GenZD to a telehealth-delivered group CBT (CBT Group) on depressive symptoms at week 4 (end of treatment [EOT]). Tertiary aims were to assess participants’ working alliance, anxiety, and general mood, and safety aims were to assess safety over the study period. Finally, potential differences in between-group treatment outcomes were explored in different race/ethnicity, gender identity, and sexual orientation categories as exploratory analyses.

## Method

This study was reviewed and approved by the WIRB-Copernicus Group (WCG) institutional review board on March 31, 2022 (#20221397).

### Participants and Procedures

The study site was an outpatient program in a free-standing children’s hospital in the southeastern United States, serving a racially and economically diverse population. The study procedures were integrated into a stepped care model of outpatient care, in which referred patients are seen for a triage appointment resulting in a preliminary clinical diagnosis and assessment of level of care needed (routine or fast track). Only youth assigned to routine level of care (no active safety concerns, no psychosis, eating disorder, bipolar disorder, primary substance use disorder, or level of impairment as indicated by 4 or more psychiatric medications or imminent risk of exclusion from school) were eligible for the study. Those who declined the study were offered telehealth CBT Group while waiting for definitive individualized care. Youth who participated in the study were still eligible for individual definitive care without any additional delays.

Adolescents with predominantly depression and/or anxiety symptoms triaged into routine level of care (routine care) were eligible to be screened for study participation. Study-specific eligibility criteria included the following: age 13 to 17 years; US residency; youth and caregiver able to read and write in English; reliable access to a smartphone and Wi-Fi or sufficient data to participate for the study’s duration; and availability to engage with the study for 8 weeks. For youth on psychiatric medication, dose(s) needed to be stable for at least 60 days prior. Caregivers agreed to engage in safety planning with study clinicians in the event that suicidal ideation developed during the study.

Exclusion criteria included the following: lifetime history of autism spectrum disorder; current diagnosis of developmental delay or intellectual disability; reported suicidal ideation with plan or intent or a suicide attempt within the past 12 months; diagnosis of substance use disorder within the past 12 months; current use of benzodiazepines or certain sleep aids (zolpidem, eszopiclone, zaleplon); previous Woebot application use; current psychotherapy participation, and study enrollment of another household member. Inclusion and exclusion criterion did not specify a lower or an upper limit on total baseline depression score, as measured by the eight-item Patient Health Questionnaire (PHQ-8).^33^

Respectively, informed assent and consent for eligible adolescents and their caregivers was obtained for participation in study procedures, including agreement to use the study help line or to seek clinical care if safety concerns arose. At randomization, youth randomized to CBT Group were scheduled for their first group date within 1 week. All W-GenZD and CBT Group intervention access concluded after 4 weeks. An 8-week assessment followed a period of 4 intervention-free weeks as the follow-up period to explore safety effects during the follow-up period. Measures were completed through an electronic data capture system, accompanied by automated text and e-mail reminders and telephonic outreach by study personnel.

To support access to definitive care, adolescents who accessed nonstudy psychotherapy were withdrawn from the study. Adolescents who changed medications and/or dose post enrollment continued participating, with the exception of those using excluded medications.

**Figure 1:**
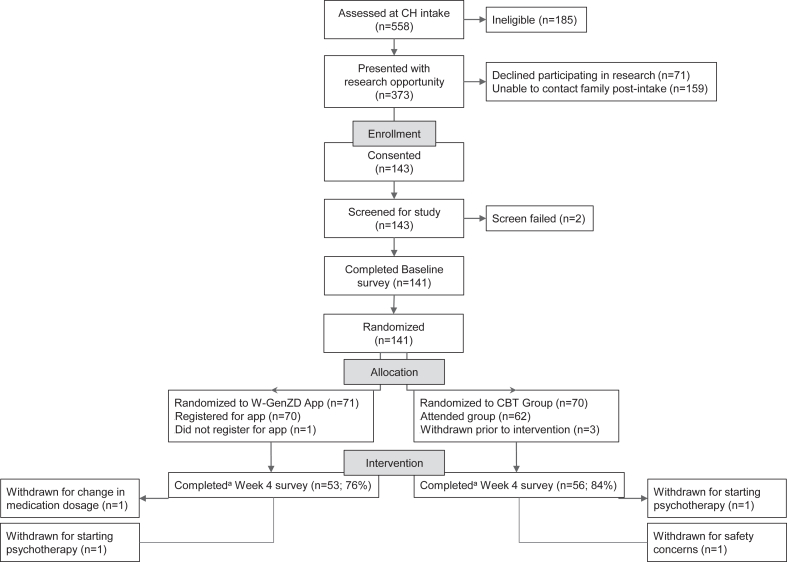
**CONSORT Diagram** ***Note:****CBT = cognitive****-****behavioral therapy; CH = children’s hospital.* ^*a*^*Completion here is defined as responding to the survey. It does not require that all items were answered, which is why participant numbers (n) may differ from those reported in the main text on missingness and complete case scoring (which required that participants responded to every item of the survey to be scored). Protocol was later modified to eliminate the study requirement to register for the W-GenZD App or to attend telehealth-delivered CBT Group within the first week of randomization.*

Figure 1 depicts participant flow through the study. Additional details of the study design and methodology are available in Chiauzzi *et al.*^34^

### Assessments

The assessment timepoints were baseline (BL) and 4-week EOT, with an additional 4-week follow-up period (8 weeks post BL) to assess safety. Measures were of demographics and psychiatric history (BL), feasibility and acceptability (EOT), depressive symptoms (BL, EOT), as well as general mood and anxiety symptoms (BL, EOT), and working alliance (EOT). Assessment reminders were delivered via e-mail and SMS with active survey links and offered up to $125 in Amazon gift cards compensation for time spent in completion of all assessments. Caregivers completed phone check-ins with the study coordinator at 2, 4, 6, and 8 weeks to identify changes in their child’s medications, or logistical or safety concerns; if the latter were identified, the safety protocol was initiated. Assessments included the elements described below.

#### Usage Rating Profile—Intervention Revised

We used a 6-item feasibility scale adapted for use by the patient rather than the provider, and developed a 6-item acceptability scale derived from the Usage Rating Profile—Intervention Revised (URP-IR) scale,^35^ both scored on a 6-point Likert scale. Subscale scores range from 6 to 36, with higher scores indicating greater feasibility and acceptability.

#### Intervention Adherence

Adherence, inclusively defined *a priori* to allow for real-world comparison, was W-GenZD use during at least 2 of the 4 treatment weeks, or CBT Group attendance at least 2 of the 4 treatment sessions.

#### Patient Health Questionnaire

The 8-item Patient Health Questionnaire (PHQ-8)^33^ is a measure scored on a 4-point Likert scale. Scores range from 0 to 24, with higher scores indicating increased severity of depressive symptoms.

#### Generalized Anxiety Disorder Questionnaire

The 7-item Generalized Anxiety Disorder questionnaire (GAD-7)^36^ is a measure scored on a 4-point Likert scale. Scores range from 0 to 21, with higher scores indicating increased severity of anxiety symptoms.

#### Mood and Feelings Questionnaire—Short Form

The Mood and Feelings Questionnaire–Short Form (MFQ-SF)^37^ is a 13-item measure scored on a 3-point Likert scale. Scores range from 0 to 26, with higher scores indicating greater depressive symptomatology.

#### Working Alliance Inventory—Short Revised

The Working Alliance Inventory—Short Revised (WAI-SR)^38^ is a 12-item measure examining the overall working alliance between the participant and the therapist The WAI-SR was used with minor adaptations (ie, replacing the word “therapist” with “Woebot” for those in the W-GenZD group, as previously described by Darcy *et al.*^32^ WAI yields an overall composite score and 3 subscales: Bond, Goal, and Task.

### Safety Monitoring

The study’s Safety Monitoring Plan included safety protocol procedures, determined *a priori* and initiated following prompts from any of the following safety monitoring methodologies from baseline through 8 weeks. These procedures included participant and/or caregiver spontaneous report to study personnel through a 24-hour hotline; phone check-ins with the caregiver; and concerning language detection within either intervention. For both intervention groups, caregiver check-in was scheduled at 2, 4, 6, and 8 weeks to maintain communication, to check in about any concerns about interval safety and any interim treatment changes, and to encourage ongoing communication. CBT Group leaders monitored for safety concerns and, if noted, prompted safety protocol follow-up procedures including with caregiver as clinically indicated. Similarly, free text inputs containing potentially concerning language by W-GenZD participants were flagged from the device’s “safety net protocol,” which alerted the on-call clinician who would reach out directly to the caregiver to assess context and initiate emergency procedures if needed (Supplement 1, available online, provides a detailed description of the W-GenZD intervention; Chiauzzi *et al.*^34^ includes a full description of W-GenZD’s safety net protocol and its associated algorithm).

Upon learning of a potential safety concern, licensed study personnel, on call to provide follow-up 24 hours a day, 7 days a week, contacted the caregiver to assess the adolescent's safety, to identify the level of intervention needed, and to provide resources as needed. Participants continued in the study if they continued to meet the study inclusion and exclusion criteria and were not deemed to require a higher level of care. Events were categorized as applicable as Adverse Events (AEs), Serious Adverse Events (SAEs) or Unanticipated Adverse Device Effects (UADEs) according to standard accepted definitions.^39^

### Interventions

CBT Group included four 60-minute sessions. Generally, each adolescent had the same group leader for their groups, with minimal exceptions (ie, illness, maternity leave). Cameras were required to be on during introductions (ie, included name, preferred pronouns, a fun fact about themselves) to foster group connections. Every session started with a mindfulness moment, psychoeducation with the thoughts**–**feelings**–**behavior triangle, and homework review to ensure a strong focus on the fundamental elements of CBT in the open groups. The bulk of the session focused on introduction of a new skill, in vivo practice, and assignment of homework. The 4 session topics included core CBT elements: identifying and addressing automatic thoughts, emotion regulation, problem solving, and behavioral activation, using core elements of CBT.^40^ Group content was adapted from a 6-session model that the children’s hospital was already deploying to a 4-session model to match previous studies of W-GenZD and to ensure that both arms of the treatment were of equal duration. The content of the sessions was restructured to match the known duration of use of the app at the time that the study was implemented, as well as based on the knowledge that diverse CBT delivery approaches, as brief as 3 hours, are effective. The 4 sessions incorporated key elements of effective CBT, and no major topic areas were eliminated from the 6-session model.^41^ Fidelity to CBT was ensured by a research team member observing groups.

W-GenZD is an investigational device, accessible as a smartphone app, available only to study participants. W-GenZD content is derived from the core theoretical tenets of evidence-based psychotherapies including CBT, Dialectical Behavior Therapy, and Interpersonal Psychotherapy. Woebot, a self-help relational agent that guides participants through the program, offers evidence-based emotion management techniques, mood tracking, empathetic responses and psychoeducation, all centered around the management of mood, depression, and anxiety symptoms. Conversations are supported by both natural language**–**processing algorithms at free-text entries as well as forced-choice button replies, with check-ins to confirm understanding and motivation for next steps. The tailoring was purposefully designed to respond to the adolescent’s real-time report of the presenting situation, to help them practice and develop emotion regulation skills in the context of their everyday life for their acknowledged problem at hand (Figure S1, available online).

W-GenZD was embedded with a device-specific safety protocol that followed safety recommendations from the American Psychiatric Association (APA)^42^ and the American Medical Association (AMA).^43^ These safety features included the following: providing the privacy policy and terms of service; informing the participant that they were talking to a relational agent (Woebot), not a real person; communications that the device was not a crisis intervention; directly encouraging users to seek additional support outside of the device; and listing helplines for additional assistance. W-GenZD’s “safety net protocol” was based on a natural language**–**processing algorithm designed to detect concerning language. The algorithm flagged text input that may contain concerning language by matching it with an *a priori* list of words and phrases. When potentially concerning language was flagged, the participant was reminded that W-GenZD was not a crisis intervention service, and the device immediately listed helplines for additional support. For this study, W-GenZD contained a specific escalation procedure: if an adolescent had concerning language flagged, they were also asked if they were in crisis, and if they answered “yes,” W-GenZD sent a Health Insurance Portability and Accountability Act (HIPAA)**–**compliant alert to designated clinical staff who reached out to the caregiver for assessment of the situation and any needed intervention. Adolescents and caregivers were informed of this W-GenZD messaging capability, and provided assent and consent accordingly.

### Statistical Methods

As the primary aims were descriptive, sample size calculations were performed for the secondary aim. Secondary analysis compared the effects of the 2 interventions on self-reported depressive symptoms (PHQ-8) at EOT using a noninferiority design. The analysis involved enrolling 260 participants (130 participants per arm), with group sample sizes of 86 and 86 (n = 172) estimated to achieve 81% power to detect noninferiority using a 1-sided, 2-sample *t* test. The margin of noninferiority was set to 2.0 points on the PHQ-8.^44^ The significance level (alpha) of the test was set at 0.025, as 1 side of a 2-sided test is needed to assess noninferiority. Prior estimates of the standard deviations, per group, were 5.3 and 4.3, based on a previous RCT of W-GenZD conducted among 16- and 17-year-olds.^30^ To account for 33% attrition, a literature-derived estimate of CBT intervention attrition,^45^ the final target recruitment sample was set to 260 to achieve targeted sample size of 172.

All statistical analyses were performed using R Statistical Software (R Foundation for Statistical Computing). Results are reported overall and for each treatment arm at each time point in the study using an intent-to-treat (ITT) approach, which included all participants randomized to the study. Partly because of a third-party platform administrative error (ie, failure of automatic reminder to participants to complete an assessment) (12/40) and participants either not completing or skipping items (28/40), 28% were missing complete EOT PHQ-8 data. Completers vs noncompleters did significantly vary by several available demographic factors (Table S1, available online), which were included in subsequent multiple imputation (MI) models to account for missingness. Specifically, to handle any item-level data missing from completed surveys because of “skipped” items, MI using other survey items as predictors was implemented. To impute entirely missing surveys, MI was used to impute scale scores with the following predictors, shown to be significantly related to outcomes in prior literature: treatment group, age, gender, education, sex at birth, race/ethnicity, sexual orientation, caregiver marital status, caregiver insurance, participant’s use of stimulants at baseline, total adherent intervention weeks, and baseline PHQ and GAD scores, with any relevant baseline scores where applicable to each specific measure. For all MI analyses, the predictive mean matching (PMM) method was implemented, 5 imputations were run, and the average across all 5 imputations was retained for analysis. MI analyses were conducted using the Multivariate Imputation by Chained Equations (MICE) package^46^ in R.

For the per protocol (PP) sample, participants with at least 1 protocol deviation were removed. Protocol deviations included the following: no longer meets eligibility criteria; not evaluable for PHQ-8 EOT survey (including missing individual items); not treatment adherent; evaluated out of assessment window at any time point; other deviation.

Descriptive summary statistics are presented about the ITT sample overall and by treatment group for feasibility and acceptability, adherence, working alliance, anxiety, and general mood. Normality of continuous variable distribution was evaluated using accepted measures of skew. The secondary and tertiary outcomes were reported also among those with elevated symptoms of depression (PHQ-8 score >9) at baseline. Means and standard deviations were reported for normally distributed variables (|skewness| ≤ 1.00), whereas medians and interquartile ranges were used for nonnormal data (|skewness| > 1.00). Descriptive statistics for safety endpoints were provided, including method of detection of potential safety concern (eg, spontaneous report), frequency, category type (eg, AE, SAE, UADE), and outcomes of potential incidents initiating the safety follow-up protocol.

A noninferiority (NI) design tested the secondary aim of determining between-group differences on reducing PHQ-measured depressive symptoms at EOT. This design compares the impact of an experimental intervention (W-GenZD) on target symptomatology to that of an active control with known efficacy (telehealth CBT Group), to determine whether the experimental intervention is no worse than the established intervention by a predetermined margin. This is done to demonstrate that the experimental intervention may be used when there is certain benefit of the experimental intervention (eg, immediate accessibility, stigma-free environment) as compared to the known treatment. Noninferiority of W-GenZD to CBT Group was declared if the upper bound of the 1-sided 97.5% confidence interval was less than 2 on PHQ-8 EOT scores. Noninferiority should ideally be claimed only when it is supported in both the ITT and PP analysis,^47^ with a recommendation to also include sensitivity analyses if possible to further bolster claims. As such, noninferiority analyses were run on the ITT sample and the PP sample, as well as sensitivity analyses controlling for baseline PHQ scores in the ITT sample, and using a complete case ITT sample (ie, restricting to those in the ITT sample with PHQ-8 scores at EOT before using MI). The multiple sensitivity analyses demonstrate that the treatment effect remains constant under different dataset configurations.

Finally, exploratory analyses investigated patterns in PHQ baseline and change scores by the following social construct characteristics potentially related to mental health: combined race**–**ethnicity, gender identity, and sexual orientation. Given the small samples by treatment group, variables were collapsed into broader subgroups (ie, Race**–**ethnicity: Hispanic or Latino, non-Hispanic Black, non-Hispanic other, non-Hispanic White; Gender identity: man/boy, woman/girl, other or prefer not to answer; male, female; sexual orientation: sexual minority/don’t know/prefer not to answer, not sexual minority). Both *t* tests and analyses of variance were used to compare baseline PHQ scores by subgroups collapsed across treatment arms, as well as change scores compared between W-GenZD and CBT Group for each subgroup. Given underpowered tests for change scores, we focused on the magnitude of effect size. These results are meant to be hypothesis generating rather than confirmatory.

## Results

Potential participants (n = 558) (Figure 1) were screened for eligibility; of these, 141 were randomized, and comprised the intent-to-treat population. The sample was predominantly female (78%), identified as non-Hispanic white (50%), heterosexual (59%), and an average age of 14.63 years. Caregivers reported mostly that they were one of the participant’s parents (94%), employed full time (69%), married (56%), and had similarly distributed health insurance between private and publicly funded insurance. Table 1 lists participant and caregiver sociodemographic characteristics, which did not differ significantly across groups.

**Table 1:** Participant and Caregiver Sociodemographic Characteristics

Sociodemographic Characteristic	Overall N = 141	W-GenZD n = 71	CBT Group n = 70	*p*^c^
	n (%)	n (%)	n (%)	
Participant age^a^, mean (SD)	14.63 (1.21)	14.55 (1.19)	14.71 (1.23)	.42
Participant race/ethnicity^a^				
Hispanic or Latino	12 (8.5)	10 (14)	2 (2.9)	.05
Non-Hispanic Black	52 (37)	27 (38)	25 (36)	
Non-Hispanic other	6 (4.3)	4 (5.6)	2 (2.9)	
Non-Hispanic White	71 (50)	30 (42)	41 (59)	
Participant sex at birth^a^				
Female	110 (78)	56 (79)	54 (77)	.84
Male	31 (22)	15 (21)	16 (23)	
Participant gender^a^				
Woman/girl	86 (61)	44 (62)	42 (60)	.23
Man/boy	39 (28)	22 (31)	17 (24)	
Other/prefer not to answer	16 (11)	5 (7)	11 (16)	
Participant sexual orientation^a^				
Not sexual minority	83 (59)	39 (55)	44 (63)	.39
Sexual minority/don’t know/prefer not to answer	58 (41)	32 (45)	26 (37)	
Caregiver relationship to participant^b^				
Legal guardian	8 (5.7)	3 (4.2)	5 (7.1)	.49
Parent	133 (94)	68 (96)	65 (93)	
Caregiver employment status^b^				
Employed (full-time or part-time)	97 (69)	49 (69)	48 (69)	1.0
Not employed/prefer not to answer	44 (31)	22 (31)	22 (31)	
Caregiver marital status^b^				
Married/partnered/cohabiting	79 (56)	36 (51)	43 (61)	.23
Divorced/separated/widowed	33 (23)	21 (30)	12 (17)	
Single/prefer not to answer	29 (21)	14 (20)	15 (21)	
Caregiver health insurance^b^				
Government insurance	68 (48)	38 (54)	30 (43)	.42
None/prefer not to answer	3 (2.1)	1 (1.4)	2 (2.9)	
Private	71 (50)	32 (45)	38 (54)	

Note: CBT = cognitive–behavioral therapy.

a Reported by adolescent participant.

b Reported by adult caregiver.

c For continuous variables, the Welch t test was conducted to compare treatment groups. For categorical variables, the Fisher test was applied to account for the small number in many cells.

The PP sample included 82 of 141 participants (58%) without any protocol deviations. Exclusion from the PP population was attributable to an incomplete PHQ-8 EOT survey (40 of 141; 28%), not meeting intervention adherence requirements (18 of 141; 13%), completing the survey out of the specified window (15 and 141; 11%), and other deviations (9 of 141; 6%). Of the participants, 59 participants had at least 1 deviation.

### Primary, Secondary, Tertiary, and Exploratory Outcomes

As shown in Table 2, feasibility and acceptability appeared descriptively similar in the 2 treatment groups. Regarding intervention adherence (Table S2, available online), 93% (66 of 71) of the W-GenZD participants used the app on at least 2 of the 4 total possible treatment weeks, whereas 81% (57 of 70) of the CBT Group participants attended at least 2 of the 4 total possible group sessions.

**Table 2:** Feasibility and Acceptability at 4-Week End of Treatment

Variable	Overall sample N = 141	W-GenZD app n = 71	CBT group n = 70
	Median (IQR)	Median (IQR)	Median (IQR)
Feasibility^a^	30.00 (3.00)	30.00 (3.00)	30.00 (3.60)
Acceptability^a^	29.00 (4.80)	28.00 (3.90)	29.10 (4.81)

Note: CBT = cognitive–behavioral therapy (telehealth-delivered); IQR = interquartile range.

a Median and IQR are reported because variables were not normally distributed.

**Figure 2:**
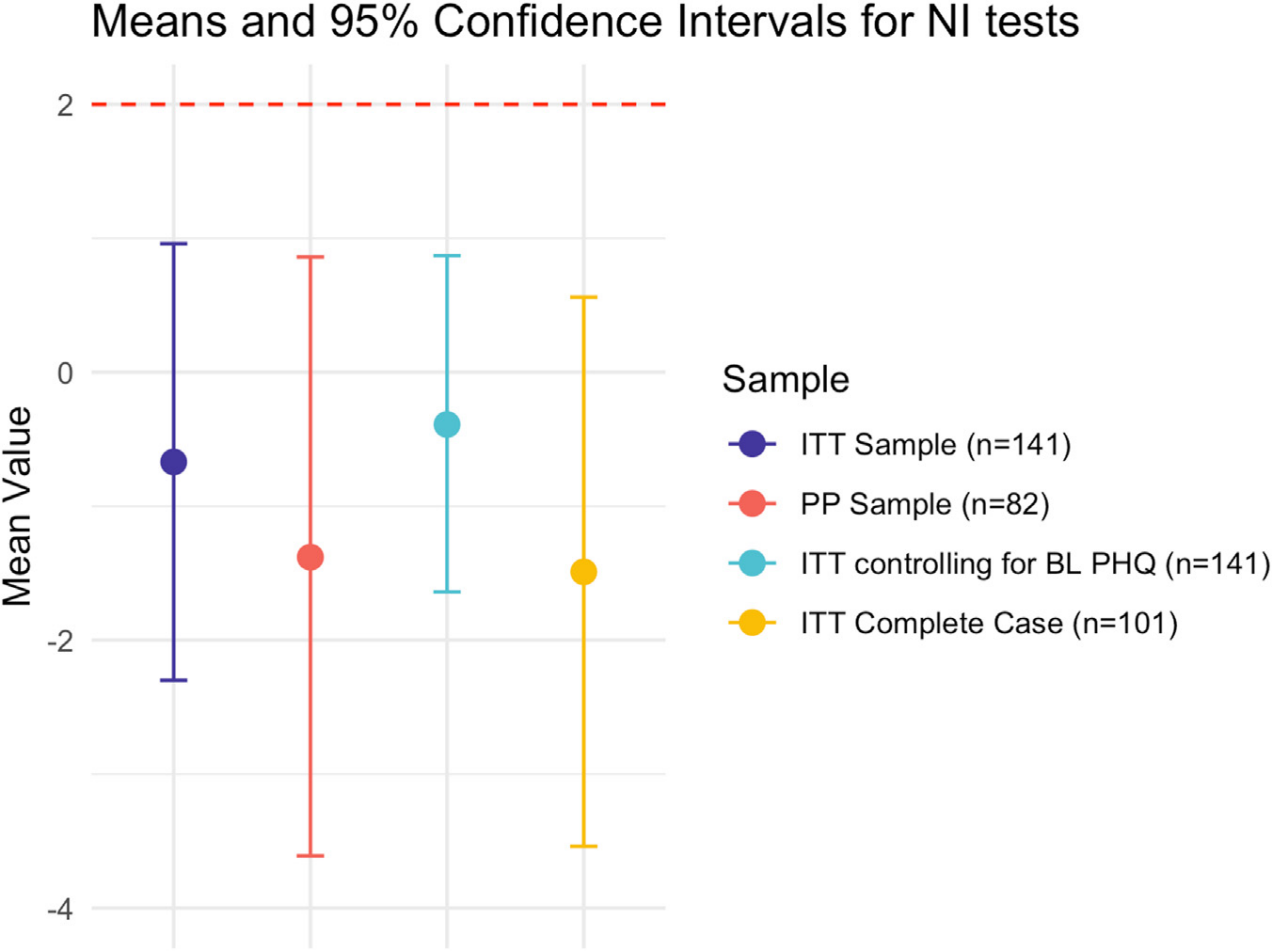
Noninferiority Results and Plot: W-GenZD and Telehealth-Delivered Cognitive**–**Behavioral Therapy (CBT) Group on Patient Health Questionnaire (8-item Patient Health Questionnaire [PHQ-8]) at 4 Weeks (End of Treatment [EOT]) ***Note:****The 95% CIs for the intent-to-treat (ITT) and per protocol (PP) sample noninferiority analyses, as well as sensitivity analyses, are displayed. The y-axis represents the between-group mean difference in EOT 8-item Patient Health Questionnaire (PHQ-8) scores. Noninferiority is declared if the 95% upper CI does not cross the vertical line at y = 2. The noninferiority margin was estimated and specified before the study started. BL = baseline.*

The entire sample’s average PHQ-8 baseline score was in the moderate severity (10-14) range and did not differ across treatment groups in the ITT, complete case, or PP samples (Table 3). Within the ITT sample, the between-group difference (W-GenZD vs CBT Group) in the mean PHQ-8 EOT scores was −0.67, 95% CI = −2.30, 0.96, and thus within the prespecified noninferiority margin of 2 (ie, the CI upper limit of 0.96 fell below 2). This pattern was replicated in the PP sample, where the mean PHQ-8 EOT score between-group difference (W-GenZD vs CBT Group) was −1.38, 95% CI = − 3.61, 0.86, again falling below the predetermined CI upper limit of 2. Two additional ITT sensitivity analyses supported the main findings within a noninferiority margin of 2: (1) controlling for baseline PHQ-8 scores demonstrated a mean PHQ-8 EOT score difference of −0.96, 95% CI = −2.75, 0.83; and (2) a complete-case ITT sensitivity analysis showed a mean PHQ-8 EOT score difference of −1.49, 95% CI = − 3.54, 0.56. Taking all findings together, these results indicate that W-GenZD is noninferior to CBT Group in its reduction of PHQ-8**–**measured depressive symptoms at EOT (Figure 2).

**Table 3:** Descriptive Summaries of Clinical and Working Alliance Outcomes by Treatment Group Among All Intent-to-Treat and Complete Case Participants as Well as Those With Clinically Elevated Depressive Symptoms (PHQ Score >9 at Baseline)

	All participants in ITT sample		Participants in complete case sample		Participants with clinical elevation^a^	
	W-GenZD n = 71 mean (SD)	CBT Group n = 70 mean (SD)	W-GenZD n = 49 mean (SD)	CBT Group n = 52 mean (SD)	W-GenZD n = 43 mean (SD)	CBT Group n = 52 mean (SD)
PHQ-8						
Baseline	10.65 (6.31)	11.16 (5.20)	10.95 (5.97)	11.76 (5.37)	14.80 (3.94)	13.23 (4.20)
Week 4	8.75 (4.91)	9.42 (4.88)	8.84 (5.28)	10.33 (5.06)	11.00 (4.72)	10.32 (4.82)
GAD-7						
Baseline	10.68 (5.87)	11.27 (5.44)	10.84 (6.03)	11.25 (5.30)	14.67 (3.26)	13.54 (4.28)
Week 4	9.11 (4.97)	9.74 (5.04)	8.81 (5.49)	9.79 (5.59)	11.23 (4.72)	10.77 (4.84)
MFQ-SF						
Baseline	11.39 (6.55)	12.87 (6.17)	11.96 (6.54)	13.58 (6.10)	14.87 (5.06)	14.73 (5.58)
Week 4	9.39 (6.71)	11.53 (6.25)	9.94 (7.14)	11.68 (6.82)	11.47 (6.91)	12.90 (6.19)
WAI-SR						
Week 4						
Goal	3.81 (0.85)	3.55 (0.92)	3.78 (0.94)	3.53 (1.02)	3.87 (0.96)	3.50 (1.00)
Task	3.26 (0.81)	3.25 (0.90)	3.23 (0.89)	3.28 (0.97)	3.21 (0.87)	3.22 (0.97)
Bond^b^	4.30 (0.85)	4.25 (0.66)	4.50 (1.00)	4.25 (0.93)	4.26 (0.95)	4.25 (1.21)
Total	3.73 (0.69)	3.63 (0.74)	3.70 (0.81)	3.65 (0.81)	3.71 (0.79)	3.59 (0.82)

Note: CBT = cognitive–behavioral therapy; GAD-7 = 7-item Generalized Anxiety Disorder questionnaire; ITT = intent-to-treat; MFQ-SF = Mood and Feelings Questionnaire—Short Form; n = number of participants; PHQ-8 = 8-item Patient Health Questionnaire; WAI-SR=Working Alliance Inventory—Short Revised; Week 4 = end of treatment [EOT].

a Symptoms of depression or anxiety were defined as clinically elevated by having a PHQ-8 score >9 or GAD-7 score >9 at baseline.

b Median and interquartile range reported because of nonnormal distribution.

Descriptive statistics for anxiety symptoms (GAD-7), overall mood (MFQ-SF), and working alliance (WAI-SR) are presented in Table 3 by treatment group, for the overall ITT sample and a subsample with elevated depressive symptomatology at baseline (PHQ-8>9). GAD-7, MFQ-SF, and WAI-SR total and subscale scores (ie, Goal, Task, and Bond) for the overall sample and the clinically elevated sample appeared descriptively similar across both groups at baseline and EOT.

As seen in Table S2, available online, there were no significant differences in baseline PHQ scores across race/ethnicity subgroups or health insurance subgroups. Girls had higher baseline PHQ scores than boys, and sexual minority participants also had significantly higher baseline PHQ scores compared to not sexual minorities. When exploring change in PHQ outcomes at week 4 by treatment group (Table S3, available online), no between**–**treatment group effects were statistically significant, although some of the exploratory findings suggest patterns indicative of potential differential treatment effects in certain sociodemographic groups. For example, between-group effect sizes suggested a possible greater reduction in the W-GenZD group as compared to the CBT group for non-Hispanic Black participants (Cohen *d* = −0.36, 95% CI = −0.90, 0.19), but similar reductions across treatment groups in the non-Hispanic White group (Cohen *d* = 0.12, 95% CI = −0.35, 0.59). There also may be a greater decrease in depressive symptoms for girls in the W-GenZD vs CBT Group participants (Cohen *d* = −0.27, 95% CI = −0.69, 0.16), but likely no differential treatment outcomes for boys (Cohen *d* = 0.03, 95% CI = −0.61, 0.66). There did not appear to be differential treatment effects, however, across sexual orientation or insurance status.

### Safety Outcomes

There were 4 total AEs, 2 in each intervention group. Both CBT Group AEs were related to worsening symptoms, one of which included nonsuicidal self-injurious behavior; 1 AE was detected via the 2-week parental check-in, and the other was spontaneously reported in the follow-up period between 4 and 8 weeks. Of the 2 W-GenZD AEs, 1 AE was related to worsening anxiety and the other to intrusive thoughts; both W-GenZD participants also spontaneously reported missing the intervention, W-GenZD, after access was removed at EOT. Both W-GenZD AEs were detected at 8-week follow-up parental check-ins. None of the events were deemed an SAE or related to their respective intervention, and none were more than of moderate severity. In addition, there were 3 instances of triggering the safety net protocol within W-GenZD via free-text inputs, all of which prompted the Safety Protocol Procedures as planned. The 3 instances included the following: (1) asking for help for a friend; (2) a request for help to ensure assessment completion gift card receipt; and (3) a misinterpreted prompt in the safety net protocol (free-text entry was “dyed” hair, which triggered safety net protocol given the word “dye”).

## Discussion

This is the first prospective RCT to establish the feasibility and acceptability of W-GenZD as an intervention for youth seeking treatment for depression and anxiety symptoms. W-GenZD participants’ feasibility and acceptability ratings suggest a high level of acceptability and feasibility, commensurate with other reports,^25^^,^^48^ and nearly 70% of participants engaged in their respective intervention each of the 4 weeks.

Importantly, this project successfully developed a novel model of safety protocol to optimize for the detection of possible safety events, inclusive of patients, clinicians, and caregivers. W-GenZD used multifaceted monitoring methodology to link directly with a clinician who contacted the caregiver and patient for a safety assessment. No serious adverse events nor unanticipated device-related events were identified, but all modes of monitoring detection across both interventions—in-app notifications, spontaneous reports, and identification in group—were used at some point during the study, highlighting the importance of multimodal tracking of adolescents’ needs. The presence of a small number of false-negative results from W-GenZD is reassuring when screening for a low-incidence, high-risk outcome. Linking an app with a human responder for youth safety provides an important methodological innovation with the growing accessibility of digital supports.

This study also demonstrated that W-GenZD was not inferior to telehealth-delivered CBT Group at reducing depressive symptoms among adolescents presenting with depressive and/or anxiety symptoms in the ITT, complete case, and PP groups. The 4-week CBT Group used in this study included core CBT topics and offered a higher comparison intervention option than inactive comparisons used in many DMHI studies.^22^ The group also offered superior access compared to most care as usual, with access to the group within 1 week of randomization, licensed clinician delivery of evidence-based core elements of CBT, and fidelity monitoring. Intentionally, this study included youth deemed appropriate for routine level of care, meaning a mild-to-moderate intensity of symptoms. This design also provided the opportunity to examine outcomes among adolescents with at least moderate symptoms compared with the whole sample, demonstrating comparable improvement in W-GenZD and CBT Group participants. Furthermore, results also demonstrated that W-GenZD and CBT Group participants had similar working alliance, anxiety, and broadly defined mood symptoms across the study period.

To our knowledge, this is the first RCT to investigate and to demonstrate comparable levels of working alliance specifically between adolescents with a telehealth CBT Group clinician and with a relational agent. Two participants voluntarily described missing “their” Woebot at the end of the study, suggesting that the power of the relational agent**–**guided CBT approach, and that, for some, a longer duration of access, may be helpful. Previous data indicate Woebot’s ability to form a working alliance with both adult^32^ and adolescent users.^30^ A growing literature is exploring the nuance and qualities of adults’ sense of connection with DMHI,^49^ recognizing that it is not a human relationship and examining the potential influence on symptoms.

Study findings, considered within the context of the diverse sample, support the generalizability of W-GenZD’s efficacy in a real-world setting. The study population is representative of the children’s hospital geographical region, where 52% of the population is White and 30% is Black.^50^ As in the general clinical population at this children’s hospital, about 40% of the sample self-identified as LGBTQ+, which is not unexpected, as LGBTQ+ youth have higher rates of depression and anxiety than heterosexual youth.^51^ Although the inclusion of representative samples of LGBTQ+ youth is a strength of the current study, our exploratory health equity analyses suggest that W-GenZD may be particularly effective for marginalized groups such as non-Hispanic Black adolescents and for girls in particular, although additional analyses powered to detect differences are warranted to confirm these patterns and to better understand potential differential treatment response across different sociodemographic groups.

Recruitment was more challenging than anticipated; only 141 adolescents were enrolled instead of the 172 target, or, accounting for anticipated attrition, 260. Anecdotally, some adolescents cited a reluctance to join a telehealth group due to “Zoom fatigue” in 2022, and 3 adolescents withdrew from the study post random assignment to telehealth CBT Group. The group comparison intervention may have been an enrollment challenge for youth with social anxiety or those who were concerned about group confidentiality; however, attendance levels suggest that those who enrolled were not affected by negative perceptions of telehealth or group. The group duration in the study was 4 sessions rather than the health system’s usual 6-session format to match the 4-week delivery timeframe previously researched for W-Gen ZD. The group content was reviewed to ensure that the 4 session groups included critical elements of CBT based on the literature.^41^ The literature demonstrates the impact of brief interventions, and dose**–**response analysis suggests that reliable change is evident in adolescents after fewer than 3 sessions.^52^ Although we are not aware of studies indicating the strength of 6 sessions over 4 sessions, we acknowledge that this decision could introduce a chance of a type 1 error. New findings from meta-analyses in adults suggest either no association or an inverse association between effectiveness and higher number of sessions.^53^^,^^54^ Assessment completion was briefly affected by an administrative programming error in the electronic data capture system to send planned, automated invitations to complete assessments.

Despite lower-than-target enrollment, the study still met the *a priori* criteria for noninferiority, further supporting the result that W-GenZD is noninferior to telehealth CBT Group. A smaller sample size increases the CI range, making it less likely that the noninferiority margin of 2 will fall outside the CI upper bound (the requirement for declaring noninferiority). Furthermore, there was consistent noninferiority support across ITT, complete case, and PP analyses, which is the most robust evidence of noninferiority.

In this sample of youth with depressive and anxiety symptoms, W-GenZD was an acceptable and feasible intervention that resulted in reductions of depressive symptoms similar to those of a brief telehealth CBT Group used in a real-world setting. The ability of W-GenZD to influence outcomes similar to a real-world, higher-resource, clinician-based, brief intervention for adolescents makes it an important element in the service array for youth with anxiety or depressive symptoms. The promise of asynchronous, self-driven support is especially valuable for youth who, like those in this study, have had a preliminary assessment or perhaps have been seen by their primary care clinician and are awaiting definitive care. W-GenZD may meaningfully add to mental health care service optionality for the thousands of adolescents waiting for care,^55^ and may potentially expand limited workforce capacity to serve youth seeking treatment for depression and/or anxiety symptoms.^56^

## CRediT authorship contribution statement

**Mary Margaret Gleason:** Writing – review & editing, Writing – original draft, Supervision, Resources, Project administration, Investigation, Conceptualization. **Megan Flom:** Writing – review & editing, Writing – original draft, Formal analysis. **Stephanie Rapoport:** Writing – review & editing, Writing – original draft, Project administration, Methodology. **Andre Williams:** Writing – review & editing, Writing – original draft, Visualization, Validation, Formal analysis, Data curation. **Autumn Birch:** Writing – review & editing, Writing – original draft, Project administration, Methodology. **Nicole K. Wells:** Writing – review & editing, Writing – original draft, Methodology, Investigation. **Valerie Forman-Hoffman:** Writing – review & editing, Writing – original draft, Formal analysis. **Athena Robinson:** Writing – review & editing, Writing – original draft, Supervision, Project administration, Methodology, Conceptualization.
