# Supplementary material for: A Relational Agent Intervention for Adolescents Seeking Mental Health Treatment: Outcomes From a Randomized Controlled Trial Within a Children’s Outpatient Hospital
Source: JAACAP Open. 2025 Feb 11;3(4):1033–45. doi: 10.1016/j.jaacop.2025.02.002 (PMC12684459; doi:10.1016/j.jaacop.2025.02.002)
Supplement: Supplementary Figure [file mmc1.docx]

Figure S1. Screenshots of W-GenZD


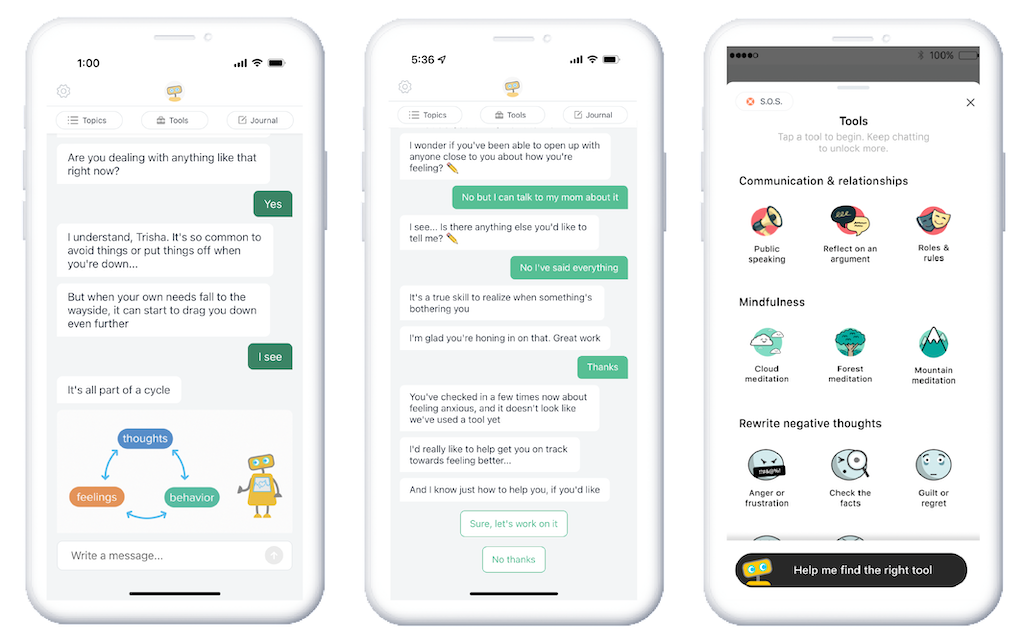
*Note.* From left to right these screenshots depict i) conversation regarding the cognitive model in cognitive behavioral therapy; ii) a conversation depicting the selective utilization of natural language processing algorithms, depicted with the invitation of free text and pencil emoji; and iii) a participants toolbox with various psychotherapeutic techniques for emotional support at the ready for in-vivo guided application practice. This figure was published and can be reproduced under the terms of the Creative Commons Attribution 4.0: Chiauzzi E, Robinson A, Martin K, Petersen C, Wells N, Williams A, Gleason MM. A Relational Agent Intervention for Adolescents Seeking Mental Health Treatment: Protocol for a Randomized Controlled Trial. JMIR Res Protoc 2023;12:e44940. doi: 10.2196/44940.
