# Supplementary material for: A Relational Agent Intervention for Adolescents Seeking Mental Health Treatment: Outcomes From a Randomized Controlled Trial Within a Children’s Outpatient Hospital
Source: JAACAP Open. 2025 Feb 11;3(4):1033–45. doi: 10.1016/j.jaacop.2025.02.002 (PMC12684459; doi:10.1016/j.jaacop.2025.02.002)
Supplement: Supplementary Material [file mmc2.docx]

**Suppl 1. Detailed description of W-GenZD**

The W-GenZD intervention is based on the core theoretical tenets of and techniques from evidence based psychotherapies e.g., Cognitive Behavioral Therapy (CBT), Dialectical Behavioral Therapy (DBT), and Interpersonal Psychotherapy (IPT). Importantly, W-GenZD is not a flat, chapter-book-like experience; it is not reading through each psychotherapy technique and moving onto the next one in queue, sequentially and without responsivity to the users’ lived experience in the moment. Instead, psychotherapeutic techniques are provided via the medium of back-and-forth text-based conversations, the flow and direction of which are in part informed by decision-tree functionality but also include thoughtfully placed natural language processing (NLP) algorithms. The utilization of these algorithms plus the decision tree format allows for ***conversational tailoring to the users inputs in real time.*** Thus, conversations can potentially vary across users because the specific therapeutic content and/or technique delivered may depend upon what the user wants to talk about and wants to work on at the time of that particular conversation. Another unique element is that W-GenZD includes Woebot; a guided self-help agent that hosts the conversations with users. Woebot, in multiple studies using a psychometrically validated measure, has repeatedly shown an ability to establish a working alliance (i.e., therapeutic bond) with the users (Darcy et al. 2021; Prochaska et al., 2021; Robinson et al.; 2022; Suharwardy et al., 2023). This is in part attributable to, via the medium of conversational flow, Woebot’s provision of a warm, welcoming, and nonjudgmental tone; use of humor when appropriate; provision of tailored empathetic statements which include reflective listening. In other words, this technology has mirrored some elements of the psychotherapeutic conversational process for rapport building, beyond content alone. Thus, Woebot is referred to as a ‘relational agent’ to distinguish it from ‘transactional chatbot agents’ designed to answer questions and/or provide flat information or services or triage, many types which are now common in the service sector (e.g., banking, airline, or hotel customer service). Woebot’s programming also stores psychotherapy techniques that the user reported as helpful, re-offering those preferred techniques as needed over time. In summary, elements that make W-GenZD unique include: conversations (e.g., therapeutic content and techniques) tailored to user input in real time; inclusion of a guided self-help agent that has reliability demonstrated an ability to establish and sustain a working alliance with users; provision of empathy statements from Woebot to the user; and ability to remember and re-offer techniques previously voted as helpful by the user.

W-GenZD was developed for ages 13-17 years with a targeted reading level to not exceed 8^th^ grade. There are not separate in-app experiences by age. W-GenZD, as stated in the manuscript, was designed for adolescents presenting with symptoms of anxiety and/or depression with an interest in emotional support.

Interested readers are referred to Chiauzzi et al (2023) for a full description of W-GenZD’s safety net protocol.

**Supplemental References**

Darcy A, Daniels J, Salinger D, Wicks P, Robinson A. Evidence of human-level bonds established with a digital conversational agent: Cross-sectional, retrospective observational study. *JMIR Form Res*. 2021;5(5):e27868. doi:10.2196/27868

Prochaska JJ, Vogel EA, Chieng A, et al. A randomized controlled trial of a therapeutic relational agent for reducing substance misuse during the COVID-19 pandemic. *Drug*

*Alcohol Depend*. 2021;227:108986. doi:10.1016/j.drugalcdep.2021.108986

Robinson A, Eaneff S, Darcy A. RCT of Woebot for adolescent depression compared to digital psychoeducation: The Headway Study. Presented at: Association for Behavioral and Cognitive Therapies (ABCT) 56th Annual Convention; November 17, 2022; New York City, NY.

Suharwardy S, Ramachandran M, Leonard SA, et al. Feasibility and impact of a mental health chatbot on postpartum mental health: A randomized controlled trial. *AJOG Glob Rep*. 2023;3(3):100165. doi:10.1016/j.xagr.2023.100165

Chiauzzi E, Robinson A, Martin K, et al. A relational agent intervention for adolescents seeking mental health treatment: Protocol for a randomized controlled trial. *JMIR Res Protoc*. 2023;12:e44940. doi:10.2196/44940
