# Supplementary material for: A Relational Agent Intervention for Adolescents Seeking Mental Health Treatment: Outcomes From a Randomized Controlled Trial Within a Children’s Outpatient Hospital
Source: JAACAP Open. 2025 Feb 11;3(4):1033–45. doi: 10.1016/j.jaacop.2025.02.002 (PMC12684459; doi:10.1016/j.jaacop.2025.02.002)
Supplement: Supplementary Table S1 [file mmc3.docx]

**Table S1. Descriptive Statistics for PHQ-8 EOT Completers vs Non-Completers**

|  | **Completers**  n=101  n(%) | **Non-Completers**  n=40  n(%) | **Statistical Significance^a^** |
| --- | --- | --- | --- |
| **Race/Ethnicity**  Hispanic or Latino  Non-Hispanic Black  Non-Hispanic Other  Non-Hispanic White | 7/101 (7)  30/101 (30)  5/101 (5)  59/101 (58) | 5/40 (13)  22/40 (55)  1/40 (2.5)  12/40 (30) | **p=0.008** |
| **Sex at birth**  Female  Male | 80/101 (79)  21/101 (21) | 30/40 (75)  10/40 (25) | p=0.750 |
| **Gender Identity**  Man/Boy  Woman/Girl  Other or Prefer not to answer | 28/101 (28)  11/101 (11)  62/101 (61) | 11/40 (28)  24/40 (60)  5/40 (13) | p=0.962 |
| **Sexual Orientation**  Not sexual minority  Sexual minority or Don’t Know or Prefer not to answer | 57/101 (56)  44/101 (44) | 26/40 (65)  14/40 (35) | p=0.458 |
| **Relationship to participant**  Legal Guardian  Parent | 6/101 (6)  95/101 (94) | 2/40 (5)  38/40 (95) | p=1.00 |
| **Caregiver employment**  Employed (FT or PT)  Not employed or Prefer not to answer | 75/101 (74)  26/101 (26) | 22/40 (55)  18/40 (45) | **p=0.043** |
| **Caregiver marital status**  Divorced / Separated / Widowed  Married / Partnered / Cohabitating  Single / Prefer not to answer | 21/101 (21)  64/101 (63)  16/101 (16) | 12/40 (30)  15/40 (38)  13/40 (33) | **p=0.016** |
| **Caregiver insurance**  Government  None or Prefer not to answer  Private | 40/101 (40)  3/101 (3)  58/101 (57) | 28/40 (70)  0/40 (0)  12/40 (30) | **p=0.003** |
| **PHQ-8 at baseline** *M (SD)* | 11.37 (5.66) | 9.72 (5.98) | p=0.139 |
| **GAD-7 at baseline** *M (SD)* | 11.05 (5.64) | 10.78 (5.75) | p=0.798 |
| **Treatment Group**  W-GenZD  CBT Group | 49/101 (49)  52/101 (51) | 22/40 (55)  18/40 (45) | p=0.612 |

**Note:** M(SD)=mean (standard deviation)

^a^For categorical variables with cells < 6 Fisher’s test was used. For categorical variables with cells > 6 Chi-square test was used. For continuous variables Welch’s t-test was used.
