# Supplementary material for: A Relational Agent Intervention for Adolescents Seeking Mental Health Treatment: Outcomes From a Randomized Controlled Trial Within a Children’s Outpatient Hospital
Source: JAACAP Open. 2025 Feb 11;3(4):1033–45. doi: 10.1016/j.jaacop.2025.02.002 (PMC12684459; doi:10.1016/j.jaacop.2025.02.002)
Supplement: Supplementary Table S2 [file mmc4.docx]

**Table S2. Intervention Adherence for W-GenZD**

| Number of Weeks App Opened At Least Once per Week | W-GenZD (n=71)  n (%) | Number of Weekly Group Sessions Attended | CBT Skills Group (n=70)  n (%) |
| --- | --- | --- | --- |
| 0 | 2/71 (2.8) | 0 | 8/70 (11) |
| 1 | 3/71 (4.2) | 1 | 5/70 (7.1) |
| 2 | 9/71 (13) | 2 | 7/70 (10) |
| 3 | 11/71 (15) | 3 | 10/70 (14) |
| 4 | 46/71 (65) | 4 | 40/70 (57) |

**Note:** CBT = Cognitive Behavioral Therapy; n = number of participants.
