# Supplementary material for: A Relational Agent Intervention for Adolescents Seeking Mental Health Treatment: Outcomes From a Randomized Controlled Trial Within a Children’s Outpatient Hospital
Source: JAACAP Open. 2025 Feb 11;3(4):1033–45. doi: 10.1016/j.jaacop.2025.02.002 (PMC12684459; doi:10.1016/j.jaacop.2025.02.002)
Supplement: Supplementary Table S3 [file mmc5.docx]

**Table S3. PHQ-8 Change Scores by Treatment Group for Sociodemographic Subgroups**

| **Race/Ethnicity** | | | |
| --- | --- | --- | --- |
| Non-Hispanic Black | | Non-Hispanic White | |
| *W-GenZD*  n=27 | *CBT*  n=25 | *W-GenZD*  n=30 | *CBT Group*  n=41 |
| -2.38 (4.21) | -0.84 (4.56) | -1.40 (4.85) | -1.92 (4.08) |
| Cohen’s d=-0.36 (-0.90, 0.19)  Mean difference=-1.53 (-3.98, 0.92) | | Cohen’s d=0.12 (-0.35, 0.59)  Mean difference=0.52 (-1.67, 2.70) | |
| **Gender Identity** | | | |
| Man/Boy | | Woman/Girl | |
| *W-GenZD*  n=22 | *CBT Group*  n=17 | *W-GenZD*  n=44 | *CBT Group*  n=42 |
| -0.76 (5.75) | -0.89 (4.67) | -2.54 (4.46) | -1.43 (4.01) |
| Cohen’s d=0.03 (-0.61, 0.66),  Mean difference=0.13 (-3.25, 3.51) | | Cohen’s d=-0.27 (-0.69, 0.16),  Mean difference=-1.11 (-2.93, 0.70) | |
| **Sexual Orientation** | | | |
| Not Sexual Minority | | Sexual Minority | |
| *W-GenZD*  n=39 | *CBT Group*  n=44 | *W-GenZD*  n=32 | *CBT Group*  n=26 |
| -0.94 (5.16) | -1.43(4.15) | -3.05 (4.18) | -2.25 (4.66) |
| Cohen’s d=0.11 (-0.32, 0.54),  Mean difference=0.49 (-1.57, 2.55) | | Cohen’s d=-0.18 (-0.70, 0.34),  Mean difference=-0.79 (-3.15, 1.56) | |
| **Health Insurance** | | | |
| Government | | Private | |
| *W-GenZD*  n=38 | *CBT Group*  n=30 | *W-GenZD*  n=32 | *CBT Group*  n=38 |
| -1.85 (4.98) | -2.79 (3.89) | -1.78 (4.70) | -1.28 (4.35) |
| Cohen’s d=0.21 (-0.27, 0.69)  Mean difference=0.94 (-1.21, 3.09) | | Cohen’s d=-0.11 (-0.58, 0.36)  Mean difference=-0.50 (-2.68, 1.68) | |

**Note:** Change scores are presented as means (standard deviations). 95% CIs are provided for mean difference estimates and Cohen's d estimates. The race/ethnicity “Other” subgroup, “None” insurance subgroup, as well as the gender identity “Other” subgroup did not have a large enough sample when splitting by treatment group for inclusion in this table. CBT = Cognitive Behavioral Therapy; n = number of participants.
